# Supplementary figures and images for: SCREENER: Streamlined collaborative learning of NER and RE model for discovering gene-disease relations
Source: PLoS One. 2023 Nov 27;18(11):e0294713. doi: 10.1371/journal.pone.0294713 (PMC10681162; doi:10.1371/journal.pone.0294713)

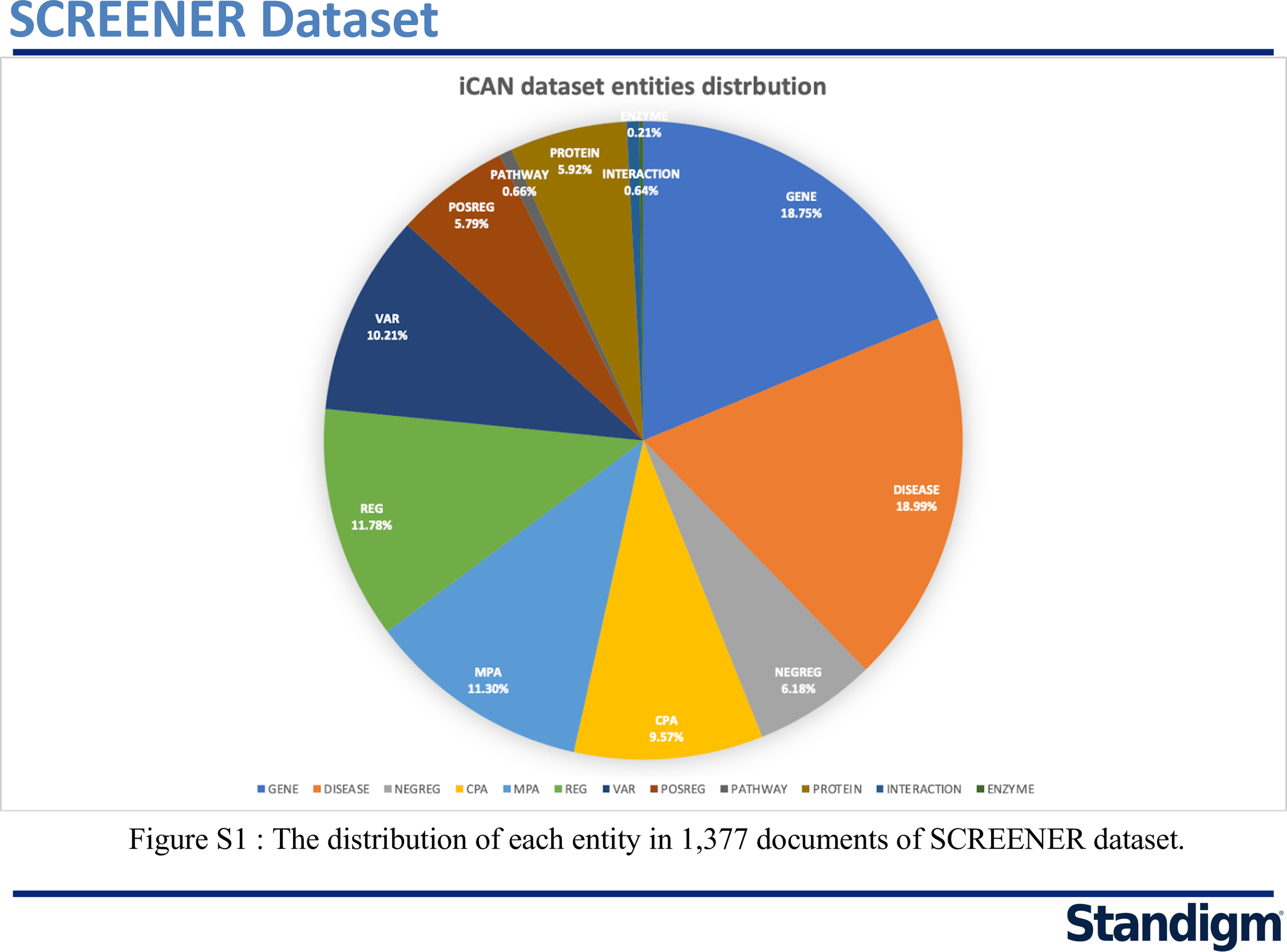

Supplement: S1 Fig — The distribution of each entity in 1,377 documents of SCREENER dataset. (TIF) [file pone.0294713.s001.tif]

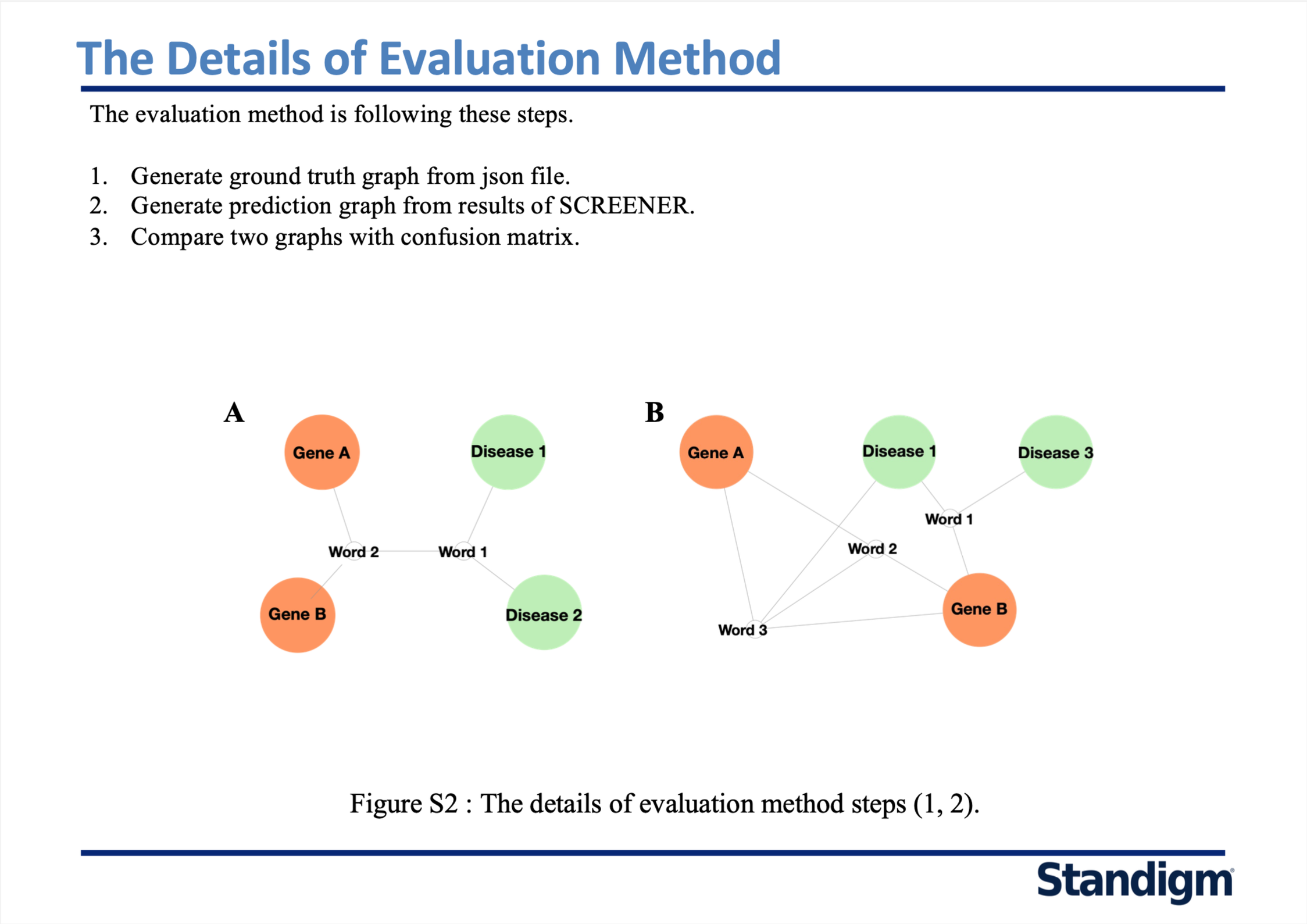

Supplement: S2 Fig — The evaluation method is following these steps. Generate ground truth graph from json file (See Fig 4A). Generate prediction graph from results of SCREENER (See Fig 4B). Compare two graphs with confusion matrix (See Fig 3). (TIF) [file pone.0294713.s002.tif]

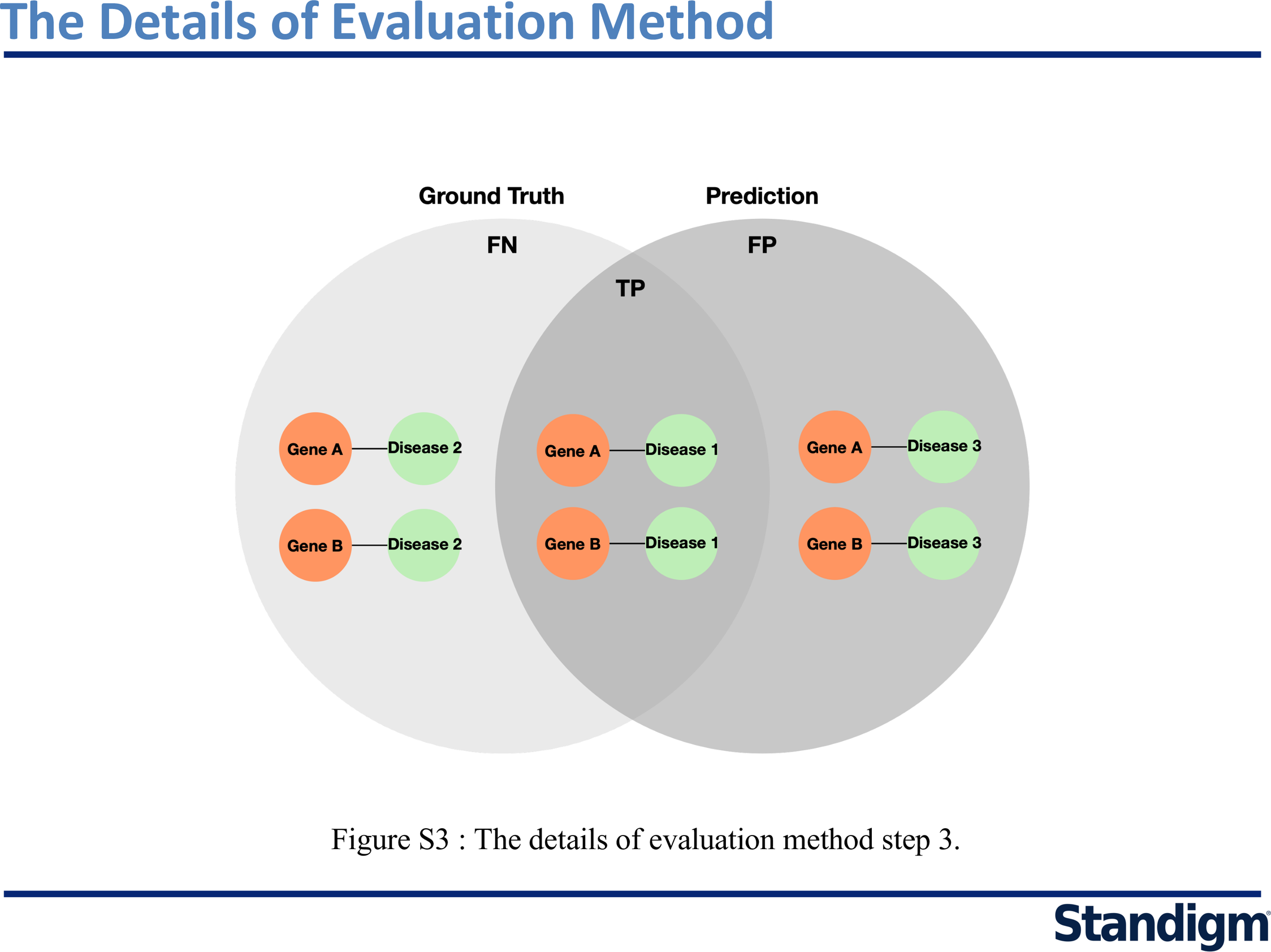

Supplement: S3 Fig — The details of evaluation method step 3. (TIF) [file pone.0294713.s003.tif]

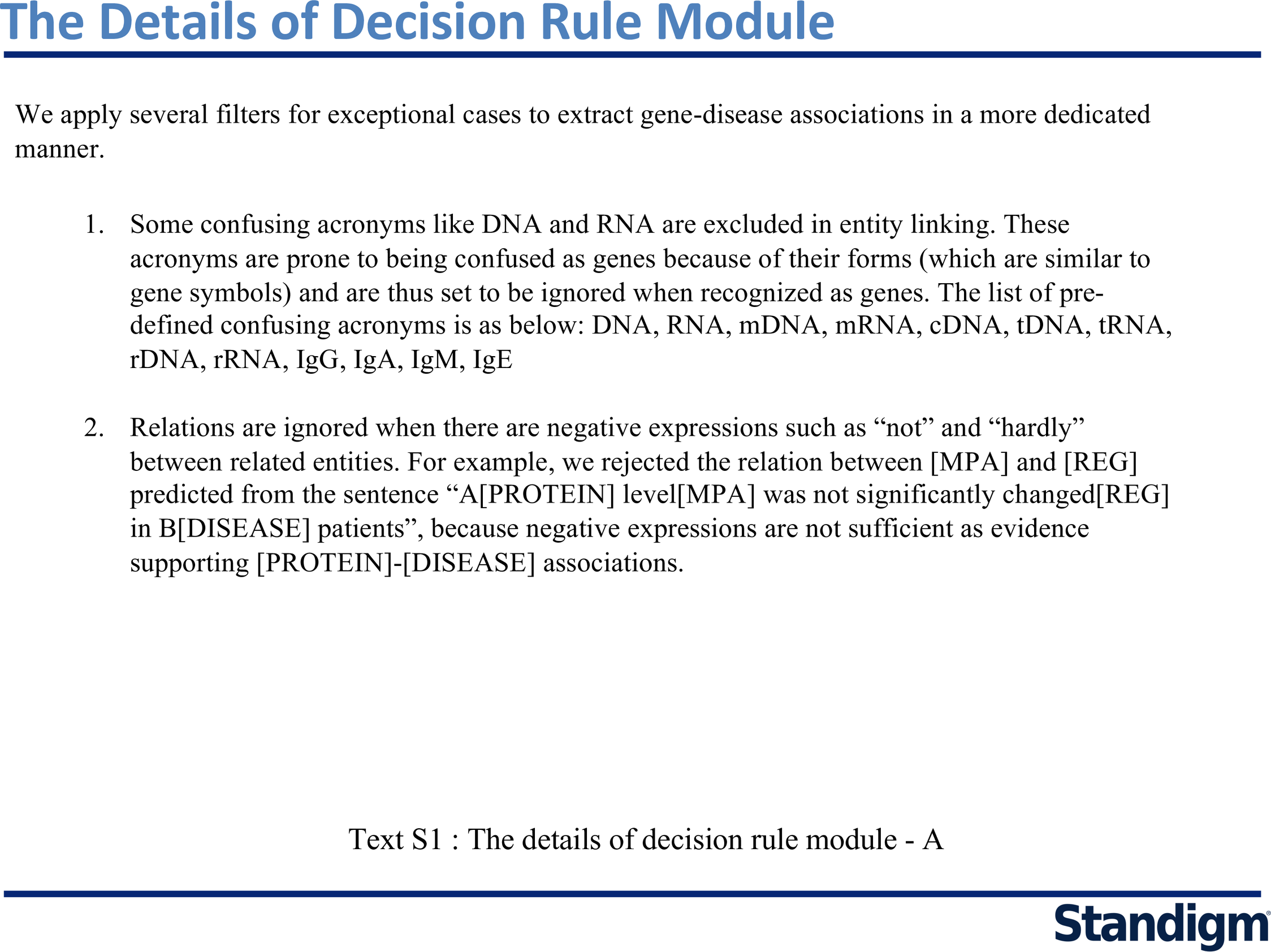

Supplement: S1 Text — We apply several filters for exceptional cases to extract gene-disease associations in a more dedicated manner. (TIF) [file pone.0294713.s004.tif]

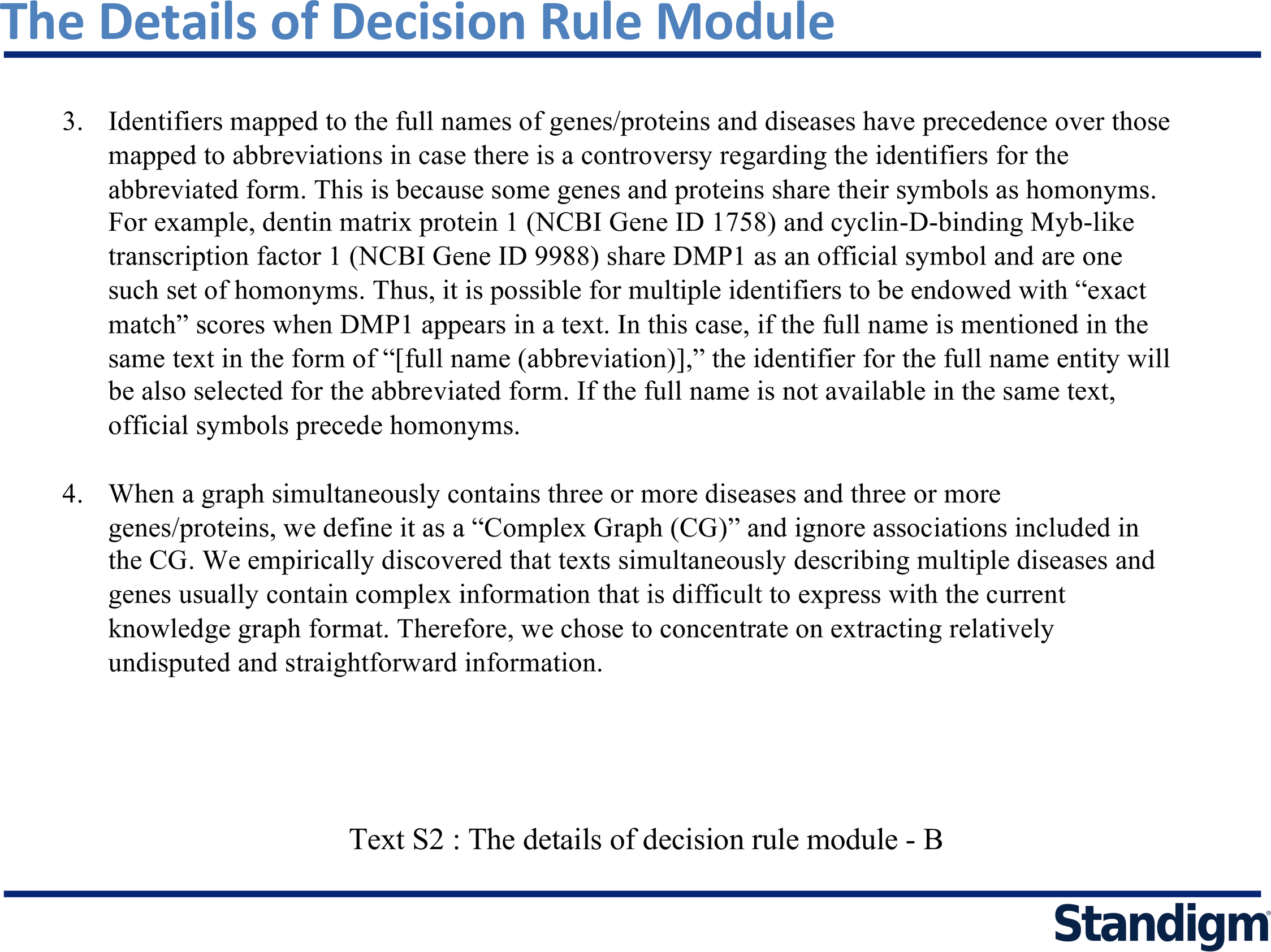

Supplement: S2 Text — The details of decision rule module—B. (TIF) [file pone.0294713.s005.tif]

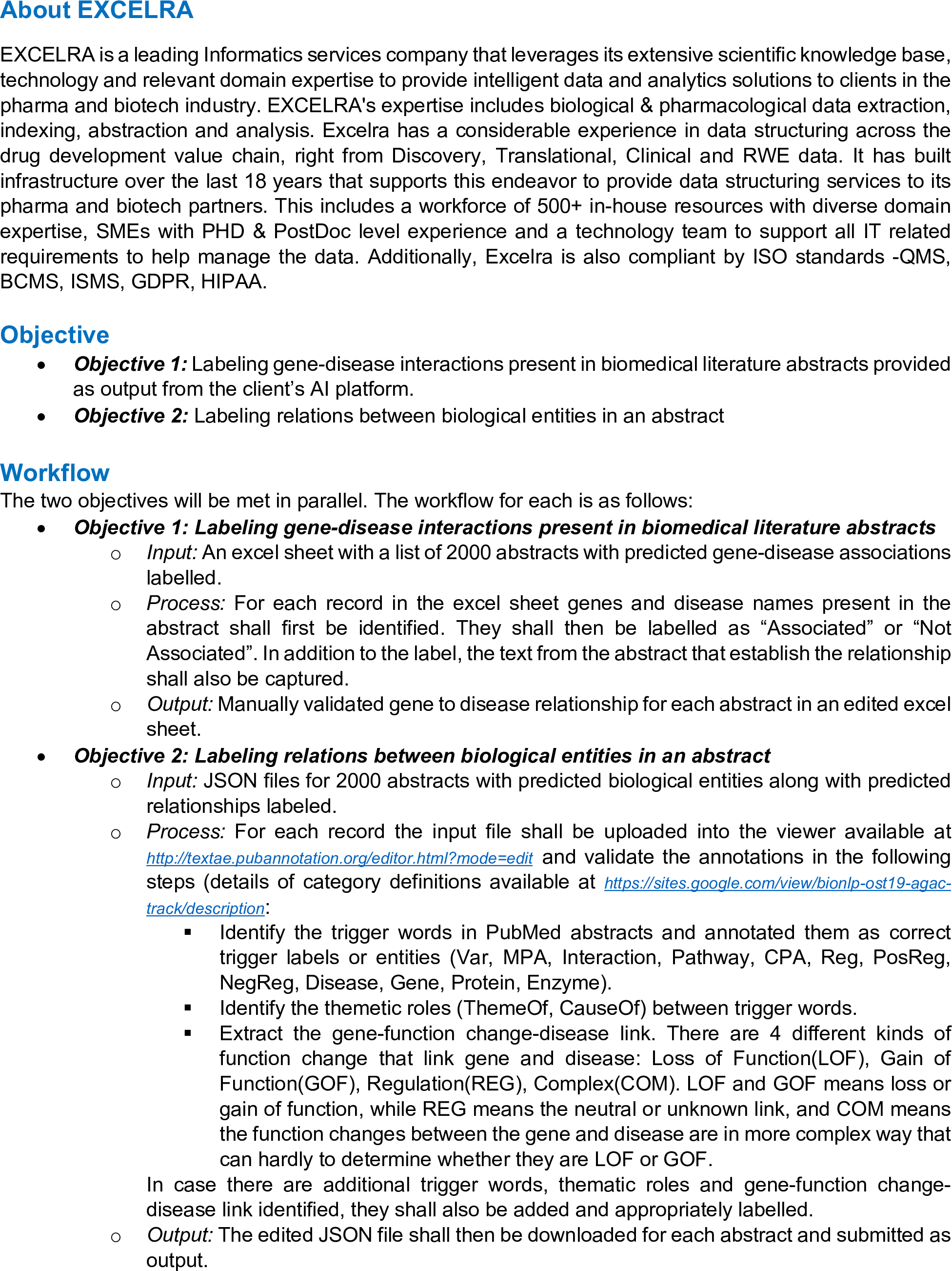

Supplement: S3 Text — A detailed data pre-processing steps described into two objectives. (TIF) [file pone.0294713.s006.tif]

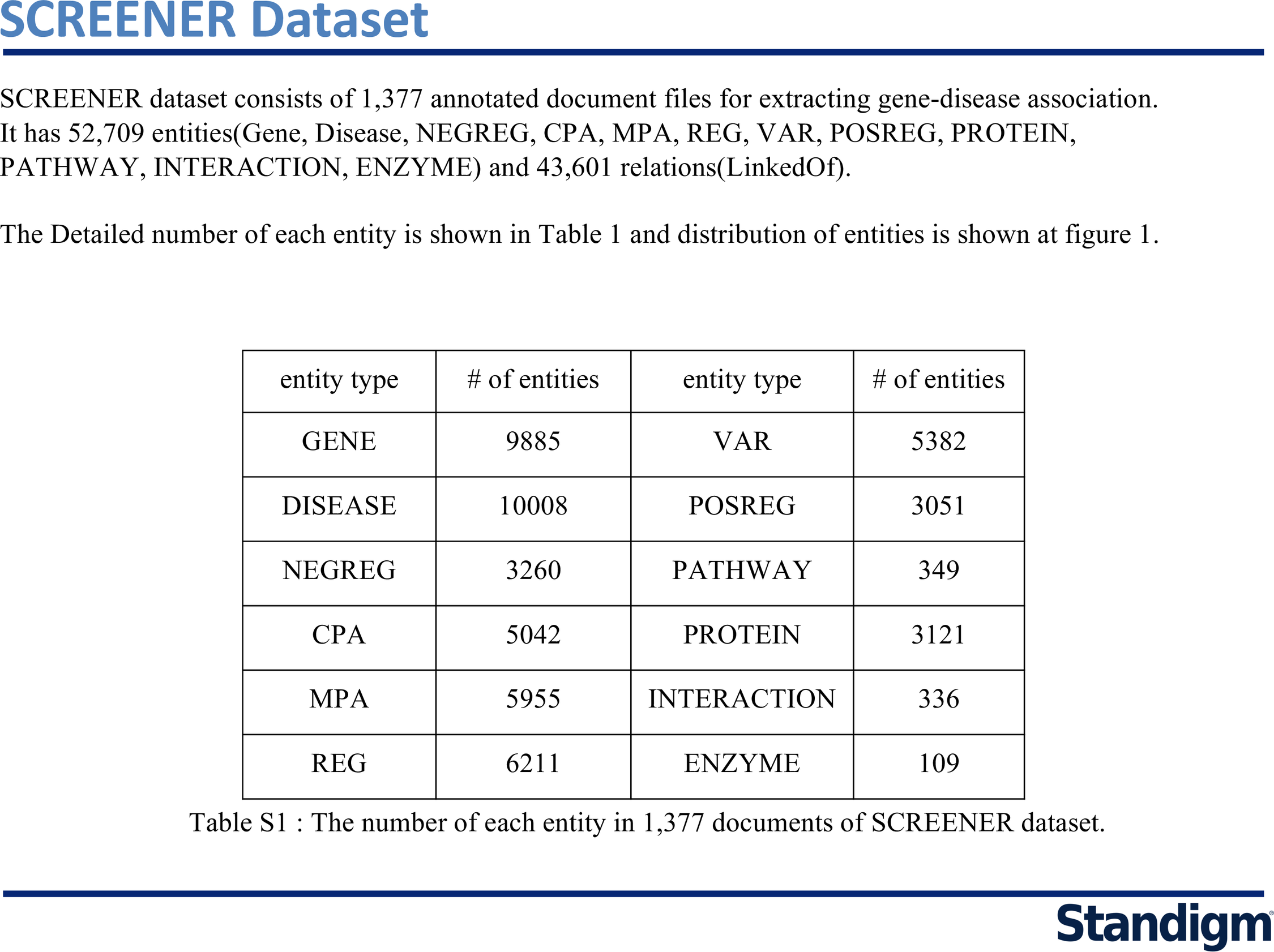

Supplement: S1 Table — SCREENER dataset consists of 1,377 annotated document files for extracting gene-disease association. It has 52,709 entities(Gene, Disease, NEGREG, CPA, MPA, REG, VAR, POSREG, PROTEIN, PATHWAY, INTERACTION, ENZYME) and 43,601 relations(LinkedOf). The Detailed number of each entity is shown in Table 1 and distribution of entities is shown in Fig 1. (TIF) [file pone.0294713.s007.tif]

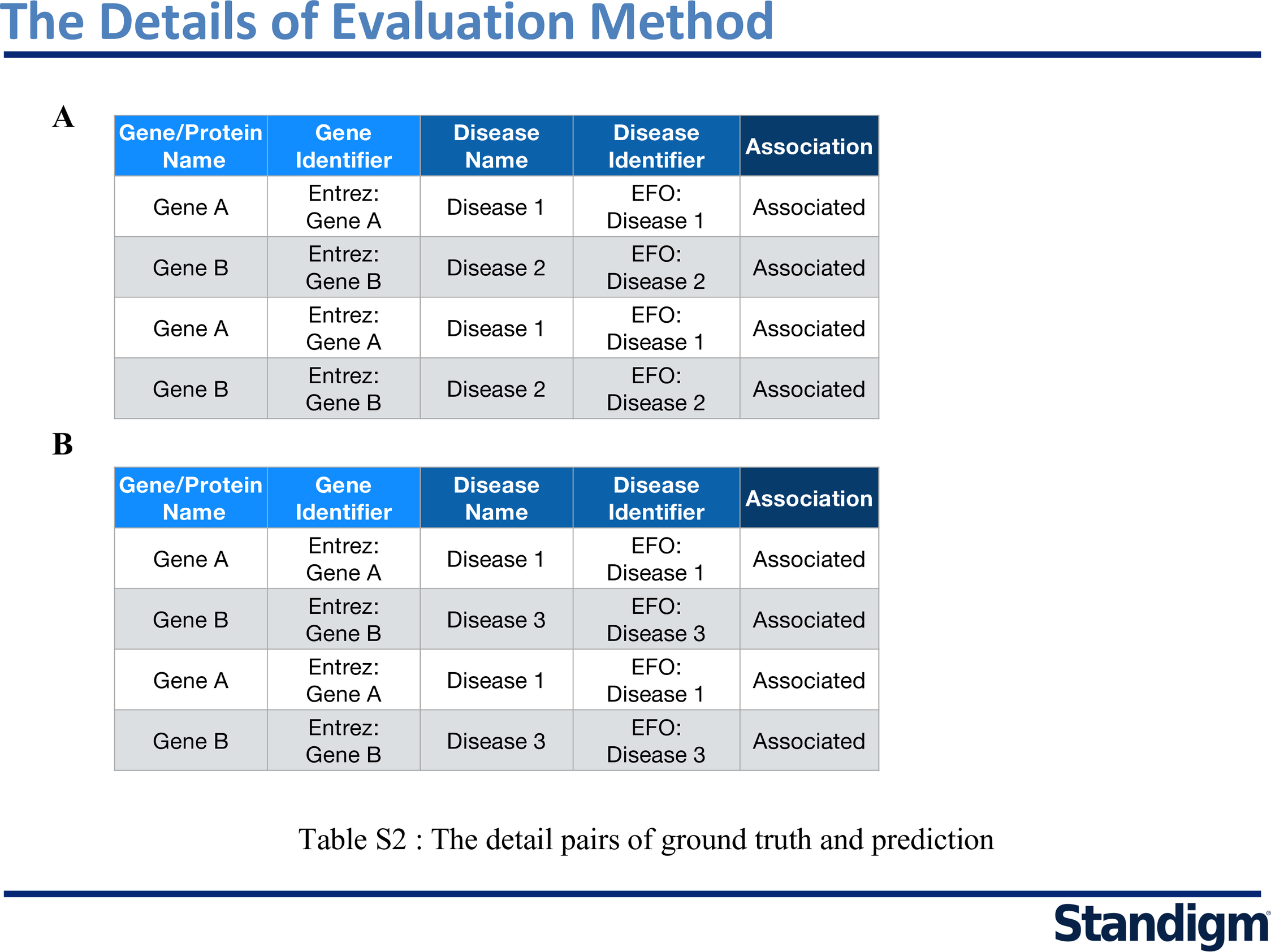

Supplement: S2 Table — The detail pairs of ground truth and prediction. (TIF) [file pone.0294713.s008.tif]
