# Supplementary material for: SCREENER: Streamlined collaborative learning of NER and RE model for discovering gene-disease relations
Source: PLoS One. 2023 Nov 27;18(11):e0294713. doi: 10.1371/journal.pone.0294713 (PMC10681162; doi:10.1371/journal.pone.0294713)
Supplement: Attachment — Submitted filename: Response to Reviewers.pdf [file pone.0294713.s010.pdf]

Point-by-point rebuttal letter

Paper: SCREENER: Streamlined Collaborative Learning of NER and RE model for discovering Gene-Disease Relations

September 27th, 2023

Reviewer #1

There is probably a mistake in the phrase "span-based tokens" in the description of Fig 2 . Usually span is the sequence of tokens, how tokens can be span-based.

We agree this was an incorrect description of span. To eliminate confusion, we modified "span-based tokens" to "span."

"annotation protocol are available at https://doi.org/10.5281/zenodo.7445644". Files have closed access, the annotation protocol is not available.

We changed to open access. The new version is available at: https://zenodo.org/record/8385406.

One of the reasons for the improvement of f1 score is stated as follows "the SCREENER utilizes trigger word enabling a richer understanding of the context". But it's unclear what does authors mean by that. If SCREENER, RENET2 and BioBERT-GAD were trained and tested on the same edges, they all used edges that connect trigger words with a disease or a gene and that way utilized trigger words. Also other methods build token embeddings considering context around it too.

To elaborate on the improvement, the novelty is application of attention mechanism on two entities predicted as Gene and Disease. In the paper, we "SCREENER applies attention mechanism on predicted gene-disease entity pairs from spans in the NER module, enabling a richer understanding of the context." In comparison, RENET2 does not apply attention mechanism, and BioBERT-GAD does not utilize spans for entity prediction.

"Detailed information on the annotation process is described in Supplemental Text 3". The archive with supplemental materials is not attached to the revision 1.

We attached Supplemental Text 3 in the revision 2. We also attached rest of supplemental files.

Reviewer #2

The only concern I have is the novelty and importance of this new approach. It is obviously an incremental improvement, but the authors should provide more arguments as to why this is worth publishing in the journal, and not a conference or workshop, for instance.

We further elaborated the novelty and importance of the SCREENER in line 394, which states, "The SCREENER is an end-to-end pipeline that learns NER and RE jointly by sharing loss. The model's novelty lies in adding a direct edge in addition to indirect edges in training. Furthermore, attention mechanism applied to predicted gene-disease pairs from spans in the NER module significantly improves the model performance with F1-score of 0.875". This should reinforce the validity of the model to be published in the journal.
